# Principal component analysis of texture features derived from FDG PET images of melanoma lesions

**DOI:** 10.1186/s40658-022-00491-x

**Published:** 2022-09-15

**Authors:** DeLeu Anne-Leen, Sathekge Machaba, Maes Alex, De Spiegeleer Bart, Beels Laurence, Sathekge Mike, Pottel Hans, Christophe Van de Wiele

**Affiliations:** 1 Department of Nuclear Medicine, AZ Groeninge, President Kennedylaan 4, 8500 Kortrijk, Belgium; 2 Department of Nuclear Medicine, University of Pretoria, Pretoria, South Africa; 3 Department of Morphology and Functional Imaging, University Hospital Leuven, Leuven, Belgium; 4 Laboratory of Drug Quality and Registration, University Ghent, Ghent, Belgium; 5 Department of Public Health and Primary Care, KU Leuven Campus KULAK Kortrtijk, Kortrijk, Belgium; 6 Department of Diagnostic Sciences, University Ghent, Ghent, Belgium

**Keywords:** Melanoma, Radiomics, LIFEx, Principal component analysis

## Abstract

**Background:**

The clinical utility of radiomics is hampered by a high correlation between the large number of features analysed which may result in the “bouncing beta” phenomenon which could in part explain why in a similar patient population texture features identified and/or cut-off values of prognostic significance differ from one study to another. Principal component analysis (PCA) is a technique for reducing the dimensionality of large datasets containing highly correlated variables, such as texture feature datasets derived from FDG PET images, increasing data interpretability whilst at the same time minimizing information loss by creating new uncorrelated variables that successively maximize variance. Here, we report on PCA of a texture feature dataset derived from 123 malignant melanoma lesions with a significant range in lesion size using the freely available LIFEx software.

**Results:**

Thirty-eight features were derived from all lesions. All features were standardized. The statistical assumptions for carrying out PCA analysis were met. Seven principal components with an eigenvalue > 1 were identified. Based on the “elbow sign” of the Scree plot, only the first five were retained. The contribution to the total variance of these components derived using Varimax rotation was, respectively, 30.6%, 23.6%, 16.1%, 7.4% and 4.1%. The components provided summarized information on the locoregional FDG distribution with an emphasis on high FDG uptake regions, contrast in FDG uptake values (steepness), tumour volume, locoregional FDG distribution with an emphasis on low FDG uptake regions and on the rapidity of changes in SUV intensity between different regions.

**Conclusions:**

PCA allowed to reduce the dataset of 38 features to a set of 5 uncorrelated new variables explaining approximately 82% of the total variance contained within the dataset. These principal components may prove more useful for multiple regression analysis considering the relatively low numbers of patients usually included in clinical trials on FDG PET texture analysis. Studies assessing the superior differential diagnostic, predictive or prognostic value of principal components derived using PCA as opposed to the initial texture features in clinical relevant settings are warranted.

## Introduction

Radiomics, the process of extracting and analysing textural features from medical images including ^18^F-fluorodeoxyglucose (FDG)/PET (positron emission tomography) CT (computed tomography) imaging, has been shown to hold promise for characterization and predicting response to treatment and outcome of human malignancies [1–3].

Many of the textural features derived by available software algorithms have proven to be highly correlated with the metabolic tumour volume (MTV) as well as to each other [4, 5]. For instance, in a study by Orlhac et al. on a group of patients suffering from various types of malignancies, using the LIFEx software, it was shown that there is no added value in calculating several indices belonging to the same group, because they describe highly correlated information. In their study, even indices from different groups were proven to still be significantly correlated [4]. Likewise, in a study by Hatt et al. specifically focusing on the relationship between entropy and dissimilarity derived from the grey-level co-occurrence matrix and high-intensity large-area emphasis and zone percentage derived from the size-zone matrix, the latter features were shown to be correlated with MTV to a different degree. The level of correlation tended to decrease substantially when larger volumes are considered [5]. Moreover, for linear models, such as multiple regression analysis, a minimum number of 10–15 patients per predictor variable has been shown to produce reasonably stable estimates [6, 7]. Thus, as a function of the number of patients under study, a specific selection of the various texture features derived through radiomics shown to be of relevance in univariate analysis, should be made for inclusion in the multiple regression analysis. To date, however, most of the clinical studies that reported on the predictive and prognostic value of texture features derived from FDG PET images have included a small number of patients and identified multiple image-derived texture features with no pre-specified analytical model which may have resulted in a statistical type-I error inflation. In a study by Chalkidou et al. [8] applying appropriate statistical corrections on a series of 15 published studies dealing with texture analysis of PET and CT studies in oncology, an average type-I error probability of 76% (range 34–99%) was estimated with the majority of published results not reaching statistical significance. Furthermore, it was suggested that the persistently high correlation identified in their study for various texture features including MTV may have led to instability of the regression coefficients weights in the multivariable model used with small changes in the data leading to very different regression coefficients [9]. Whilst some studies corrected for this phenomenon better known as “the bouncing beta’s”, this was not the case for most of the studies reported. Both phenomena could explain in part why in a similar patient population, e.g. colorectal or oesophageal carcinoma, texture features identified and/or cut-off values of prognostic significance differ from one study to another.

Principal component analysis (PCA) is a technique for reducing the dimensionality of large datasets containing highly correlated variables, such as texture feature datasets derived from FDG PET images, increasing data interpretability whilst at the same time minimizing information loss by creating new uncorrelated (orthogonal) variables that successively maximize variance. Given that the new variables are uncorrelated, PCA omits the “bouncing beta” phenomenon. Furthermore, the limited number of new uncorrelated features generated by PCA may prove more useful for multiple regression analysis when considering the relatively low numbers of patients usually included in clinical trials on FDG PET texture analysis. Here, we report on PCA of a texture feature dataset derived from 123 malignant melanoma lesions with a significant range in lesion size using the freely available LIFEx software.

## Patients and methods

### Patients

This retrospective study was approved by the ethics committee of the AZ Groeninge Hospital. The requirement to obtain informed consent was waived. Twenty-six patients suffering from malignant melanoma referred for 18F-FDG PET/CT imaging were included in the study. There were 12 men and 14 women. Mean number of lesions per patient included was 4 (range 2–10). The total number of lesions studied was 123.

### Data acquisition, reconstruction and tumour segmentation

All patients underwent a whole-body FDG PET/CT scan using a GE 64 mCT scanner. Patients fasted for at least 8 h prior to imaging to ensure a serum glucose level less than 200 mmol/L. The time difference between injection and acquisition was 60 ± 7 min following injection of 7 MBq/k body weight of 18F-FDG. PET raw data (list mode acquisition) were acquired for 1 min per bed position from the top of the skull to the proximal third of the femora or to the toes, depending on the location of the primary treated tumour. CT was performed with a tube voltage of 120 kV and a tube current ranging from 80 to 180 mAs (automatic setting). PET images were reconstructed using time of flight (TOF), point spread function (PSF) correction (QCLEAR) and a 256 × 256 matrix (corresponding voxel volume 2.7 × 2.7 × 2.7 cm^3^). SUV was calculated as 18F-FDG uptake with decay correction normalized to injected dose and patient body weight.

Tumour volumes of interest (VOIs) were delineated using region growing and a fixed threshold set to 40% of the SUVmax (standardized uptake value) in the lesions. If necessary, a manual adjustment to exclude neighbouring interfering activity was made per VOI. Tumour VOIs were delineated on the QCLEAR generated images given that they were shown to produce better image quality in terms of signal-to-noise ratio, contrast and lesion detectability. The minimal lesion volume included for subsequent analysis was 5 cm^3^.

### Texture analysis

First- and higher-order features were obtained using the IBSI-compliant LIFEx software [10–12]. Higher-order features were calculated using the grey-level co-occurrence matrix (GLCM), the neighbourhood grey-level different matrix (NGLDM), the grey-level run length matrix (GLRLM) and the grey-level size zone matrix (GLSZM). Image noise was reduced by resampling original PET values to 64 Gy levels or bins (fixed bin number discretization). A quantization of 64 Gy levels was previously shown to provide the best compromise between sufficient sampling of voxel SUVs, preservation of original intensity information and potential complementary information with respect to metabolic tumour volume [5]. In total, 38 features were studied, respectively, volume, sphericity, compacity, homogeneity, HISENT_log 10 (entropy from the histogram) and HISENT_log2, features derived from the GLCM (homogeneity, energy, contrast, correlation, entropy and dissimilarity), features derived from the GLRLM (SRE (short-run emphasis), LRE (long-run emphasis), LGRE (low grey-level run emphasis), HGRE (high grey-level run emphasis), SRLGE (short-run low grey-level emphasis), SRHGE (short-run high grey-level emphasis), LRLGE (long-run low grey-level emphasis), LRHGE(long-run high grey-level emphasis), GLNUr (grey-level non-uniformity for run), RLNU (run length non-uniformity) and RP (run percentage)), features derived from the NGLDM (coarseness, contrast and busyness), features derived from the GLZLM (SZE (short-zone emphasis), LZE (long-zone emphasis), LGZE (low grey-level zone emphasis), HGZE (high grey-level zone emphasis), SZLGE (short-zone low grey-level emphasis), SZHGE (short-zone high grey-level emphasis), LZLGE (long-zone low grey-level emphasis), LZHGE (long-zone high grey-level emphasis), GLNUz (grey-level non-uniformity for zone), ZLNU (zone length non-uniformity) and ZP (zone percentage)).

### Statistical analysis

Statistical analysis was performed using SPSS version 27. Prior to analysis, all texture features were standardized ((texture feature result – texture feature mean)/texture feature standard deviation) yielding a mean value of 0 and a standard deviation of 1 for all texture features. Standardization was performed as to make sure all the variables included have the same standard deviation and thus also the same weight, allowing for correct axis calculation of the principal components. Thus standardized texture feature data were used for principal component analysis (PCA).

The Kaiser–Meyer–Olkin (KMO) test was used to assess the suitability of the data set for factor analysis (a value > 0.6 was deemed significant). Bartlett’s test of sphericity was used to assess whether the correlation matrix of the normalized texture features proved significantly different from an identity matrix in which correlations between variables are all zero. (A *p* value < 0.05 was deemed significant.) Varimax rotation was used to maximize the sum of the variance of the squared loadings (where “loadings” means correlations between variables and principal components). It does so by creating new uncorrelated or orthogonal variables, called principal components, that successfully maximize variance. Finding such new variables reduces to solving an eigenvalue/eigenvector problem.

Principal components with eigenvalues greater than 1 (Kaiser criterion) were considered significant.

The commonalities for each principal component (the squared multiple correlations between the newly generated principal components and all other texture features) were considered significant when higher than or equal to 0.60.

## Results

Mean lesion volume was 61.9 cm^3^ (range 5–635 cm^3^).

The KMO measure of adequacy was 0.712 and Bartlett’s test yielded a p value of 0.0001, thus meeting the statistical assumptions for carrying out principal component analysis.

Seven principal components with an eigenvalue > 1 were identified. Based on the “elbow sign” of the Scree plot, the first five were retained.

The contribution to the total variance of these five principal components derived using Varimax rotation was, respectively, 30.6%, 23.6%, 16.1%, 7.4% and 4.1%. These five principal components together thus explained approximately 82% of the total cumulative variance (see Table 1).

**Table 1:** Total variance explained by the principal components

Principal component	Eigenvalue	% Variance	Cumulative % variance
1	11.3	30.6	30.6
2	8.7	23.6	54.2
3	5.9	16.1	70.3
4	2.7	7.4	77.7
5	1.5	4.1	81.8

The correlation of each principal component with the standardized texture features is shown in Table 2 (rotated component matrix; only correlations > 0.6 are reported).

**Table 2:** Principal component loadings (correlations between standardized (s) texture features and principal components; *r*-values are given between brackets)

PC1	PC2	PC3	PC4	PC5
sGLRLM_LRE (0.977) sGLRLM_LRHGE(0.971) sGLZLM_LZHGE(0.961) sGLZLM_LZE(0.947) sGLCM_Energy(0.946) sUniformity(0.833) sGLRLM_SRE(0.714) sGLRLM_RP(0.614)	sGLCM_Contrast (0.903) sGLCM_Dissimilarity (0.898) sNGLDM_Contrast(0.864) sGLZLM_SZE(0.817) sGLRLM_SRHGE(0.785) sGLZLM_HGZE(0.731) sGLZLM_SZHGE(0.726) sGLZLM_ZP(0.671) sGLRLM_HGRE(0.654)	sGLZLM_GLNUz(0.968) sGLRLM_RLNU(0.961) sGLZLM_ZLNU(0.890) sVolume(0.881) sCompacity(0.835)	sGLRLM_LGRE(0.948) sGLRLM_SRGLE(0.912 sGLRLM_LRLGE(0.870) sGLZLM_LGZE(0.677)	sGLZLM_LZLGE(0.835) sGLRLM_GLNUr(0.736) sNGLDM_busyness(0.677)

The first principal component proved highly positively correlated with sGLRLM_LRE, sGLRLM_LRHGE, sGLZLM_LZHGE, sGLZLM_LZE and sGLCM_Energy (*r* ≥ 0.946).

The second principal component proved highly positively correlated with sGLCM_contrast and sNGLDM_contrast, sGLCM_dissimilarity, sGLZLM_SZE, sGLRLM_SRHGLE, sGLZLM_HGZE, sGLZLM_SZHGE, sGLZLMZP and sGLRLM_HGRE (*r* ≥ 0.654).

The third principal component proved highly significantly positively correlated with sGLZLM_GLNU, sGLRLM_RLNU, sGLZLM_ZLNU, sVolume and sCompacity (*r* ≥ 0.835).

The fourth principal component proved highly positively correlated with sGLRLM_LGRE, sGLRLM_SRGLE, sGLRLM_LRLGE and sGLZLM_LGZE (*r* ≥ 0.677).

Finally, the fifth component proved highly positively correlated with sGLZLM_LZLGE, sGLRLM_GLNU and sGLDLM busyness (*r* ≥ 0.677).

## Discussion

In the study presented, tumour volume delineation was performed using a 40% threshold region growing method, given that previous studies have shown that a fixed threshold of 40% best approximates tumour volume [13]. Whilst a gradient-based method would have allowed assessment of the entire tumour, including areas of necrosis, they are not widely available and currently their use is mainly limited to those research centres where they were developed [14–17].

Volumes smaller than 5 cm^3^ were not included for analysis for two reasons. First, as shown previously, discontinuities such as sharp changes in image contrast when approximated by a Fourier series will be truncated with an overshoot at the border of discontinuity. This will lead to an overestimation of SUVmax values for lesions below 22 mm of diameter (or a corresponding volume of 5 cm^3^) when using the QCLEAR algorithm provided by GE, adopted in the current study, and to an underestimation of the tumour volume when using region growing for these smaller lesions [18]. Second, given that some texture parameters are based on series of neighbouring voxel values in the *x*, *y* or *z* directions and series less than 4 voxels would not make the calculations meaningful, calling for texture calculation in volumes of at least 4 × 4 × 4 = 64 voxels corresponding to a minimal volume required of at least 4 cm^3^ when using a voxel size of 4 mm and assuming sphere-like lesions [4, 19].

Using principal component analysis, the dataset of 38 texture features generated could be compressed to a dataset of 5 new uncorrelated variables or principal components that explained approximately 82% of the total variance. The first principal component, accounting for 30.6% of the total variance, proved significantly correlated with those features assessing the distribution of long homogenous runs and zones in the tumour volume with an emphasis on those with high grey levels or accordingly high SUV values. Thus, this marker likely captures the locoregional FDG distribution within the tumour emphasizing the importance of those regions with high FDG-uptake, known to be more aggressive. SUVmax values have been previously confirmed to be a significant indicator of tumour aggressiveness and prognosis in a wide variety of human malignancies, e.g. non-small cell lung carcinoma, breast carcinoma and endometrial carcinoma [20–24]. However, whilst clinically useful, a single SUV value cannot capture all of the relevant information within the tumour. Assessing tumour heterogeneity in SUV values in non-small cell lung carcinoma patients, the latter was found to be an independent predictor of overall survival in NSCLC cancer patients in multivariable analysis in a study by Hughes et al. [25]. In their study, tumour heterogeneity was evaluated as the percentage variance unexplained in the tumour region-of-interest uptake values using an ellipsoidally contoured model and a homogenous tumour mass whose voxel intensity is greatest at the centre and diminishes in a monotone fashion as one moves radially towards the periphery of the tumour for comparison. Runs and zones of different FDG uptake likely reflect cell populations with different growth rates, vascularity, necrosis and cavitation and thus different levels of aggressiveness, all of which contribute to the overall aggressiveness of the tumour [26].

The second principal component, accounting for 23.6% of the total variance, proved most correlated with those texture features assessing the contrast in the tumour volume. Contrast reflects the sharpness of the PET images and the depth of the texture grooves of SUV peaks.

The third principal component, accounting for an additional 16.1% of the total variance, proved significantly correlated with tumour volume and thus by definition also to tumour compactness (tumour volume/tumour surface area^3/2^) and to texture features assessing the non-uniformity of the length and zones of SUV levels. The latter features are known to increase when tumour volume increases as evidenced by the strong collinearity between these features and tumour volume reported previously by other authors, also including CT-imaging, and confirmed in this study [4, 5, 27]. Additionally, as shown by Welch et al. [28] using CT images obtained from head and neck and lung carcinoma patients, when correcting these features for tumour volume, e.g. GLNU, their prognostic accuracy is decreased emphasizing the importance of the tumour volume as prognosticator [28].

The fourth principal component, accounting for 7.4% of the total variance, proved highly correlated with texture features assessing the distribution of low grey level/SUV runs, both short and long. The higher the contribution of these texture features, the higher the proportion of the total tumour volume that is non-aggressive is likely to be. The degree of glucose uptake by human malignancies as imaged by FDG-PET has been previously shown to be directly related to histologic measures of tumour differentiation with well-differentiated tumours having low FDG uptake and moderately and poorly differentiated tumours having higher uptake [29, 30]. At the molecular level, a comparison of metabolism- and stem-cell-related gene expression performed by Riester et al. on a series of 552 cancer specimens derived from patients with various malignancies showed that carbohydrate/pentose/nucleotide synthesis-related genes were elevated only in tumours that had high glucose uptake, as evidenced by FDG PET imaging, and were similar in gene expression patterns to stem cells [31].

Finally, the fifth principal component proved correlated with those features assessing the rapidity of changes in intensity (SUV values) between different neighbourhoods.

Of interest, in this series, features representing randomness and entropy such as nGLCM entropy or entropy derived from the histogram proved less significantly correlated with either of the principal components obtained (*r* < 0.58) as opposed to various other features (see Table 1). Various clinical studies have previously shown features assessing entropy derived from FDG PET images to be predictive for outcome in a wide variety of human malignancies, e.g. oesophageal carcinoma and cervical carcinoma [32, 33]. Furthermore, these features proved to be highly reproducible and robust to the delineation method used [34].

### Shortcomings

This retrospective study included melanoma lesions known to be highly aggressive. It is not to be excluded that principal component analysis of other types of human malignant lesions who are less aggressive results in a different set of principal components.

## Conclusions

In this study on a series of 123 malignant melanoma lesions with a wide range in lesion size, PCA allowed to reduce the dataset of 38 texture features derived using the LIFEx software to a set of 5 uncorrelated new variables, whilst maintaining approximately 82% of the total variance contained within the dataset. These 5 new uncorrelated variables provide summarized information on the locoregional FDG distribution with an emphasis on high FDG uptake regions, contrast in FDG uptake values (steepness), tumour volume, locoregional FDG distribution with an emphasis on low FDG uptake regions and on the rapidity of changes in SUV intensity between different regions. Assessment of the clinical superiority of these new uncorrelated variables as opposed to the initial dataset of texture features in clinical relevant settings, either as a differential diagnostic tool (e.g. for separating benign from malignant lesions) or as predictors of response to treatment and outcome, is ongoing.

## Data Availability

The dataset used and analysed during this study is available from the corresponding author on reasonable request.

## Abbreviations

FDG: ^18^F-fluorodeoxyglucose
PET: Positron emission tomography
CT: Computed tomography
MTV: Metabolic tumour volume
PCA: Principal component analysis
TOF: Time of flight
PSF: Point spread function
VOI: Volume of interest
SUV: Standardized uptake value
GLCM: Grey-level co-occurrence matrix
GLRLM: Grey-level run length matrix
GLSZM: Grey-level size zone matrix
SRE: Short-run emphasis
LRE: Long-run emphasis
LGRE: Low grey-level run emphasis
HGRE: High grey-level run emphasis
SRLGE: Short-run low grey-level emphasis
SRHGE: Short-run high grey-level emphasis
LRLGE: Long-run low grey-level emphasis
LRHGE: Long-run high grey-level emphasis
GLNUr: Grey-level non-uniformity for run
RLNU: Run length non-uniformity
RP: Run percentage
SZE: Short-zone emphasis
LZE: Long-zone emphasis
LGZE: Low grey-level zone emphasis
HGZE: High grey-level zone emphasis
SZLGE: Short-zone low grey-level emphasis
SZHGE: Short-zone high grey-level emphasis
LZLGE: Long-zone low grey-level emphasis
LZHGE: Long-zone high grey-level emphasis
GLNUz: Grey-level non-uniformity for zone
ZLNU: Zone length non-uniformity
ZP: Zone percentage
